# Identity formation and mental health challenges among marginalized Chinese international students in online communities

**DOI:** 10.3389/fpsyt.2025.1464625

**Published:** 2025-03-28

**Authors:** Ruining Jin, Xiao Wang

**Affiliations:** ^1^ Institute of Higher Education, Beijing University of Technology, Beijing, China; ^2^ Civil, Commercial and Economic Law School, China University of Political Science and Law, Beijing, China; ^3^ Department of Psychology, University of Macau, Taipa, Macau SAR, China

**Keywords:** Chinese international students, marginalization, acculturative stress, online communities, mental health, coping strategies

## Abstract

**Introduction:**

This qualitative study investigates how Chinese international students adopt marginalization as a coping strategy during the acculturation process, drawing on Self-Determination Theory to examine the underlying psychological needs for autonomy, competence, and relatedness.

**Methods:**

Semi-structured interviews were conducted via social media voice chat with 12 Chinese international students, enabling an in-depth exploration of their experiences and the formation of distinct identity clusters within online communities.

**Results:**

Analysis revealed three distinct identity clusters: Gamers, who engage in online gaming to regain competence and social validation; Bachelors, who experience romantic rejection, low self-esteem, and social withdrawal, often substituting intimacy with online pornography; and Dissidents, who adopt a “double dissident” political identity marked by severe political depression, social alienation, and suicidal ideations due to censorship and fear of repercussions.

**Discussion:**

The findings underscore the urgent need for culturally sensitive mental health support and the development of inclusive online platforms that empower marginalized immigrant populations, highlighting the complex interplay between identity formation and coping mechanisms in the digital age.

## 1 Introduction

### 1.1 Marginalization and its negative impacts on the mental health of immigrants

Marginalization, per the *Cambridge Dictionary*, refers to “the act of treating someone or something as if they are not important” (1). From a sociological perspective, marginalization means the process of relegating individuals or groups to a lower or peripheral status, effectively excluding them from the benefits and opportunities available in society (2). Through a psychological lens, marginalized individuals often experience powerlessness, ignorance, poverty, illness, and insecurity (3), which would lead to psychological distress, including feelings of alienation and social exclusion (4).

In the current landscape of international education and immigration, marginalization is one of the coping strategies for sojourners and migrants to cope with acculturative stress—a term referring to the mental and psychological distresses during the process of cultural and psychological transformation (5, 6). Through the coping strategy of marginalization, individuals reject both their original culture in the home country and the new culture in the host country (5). Other coping strategies for acculturative stress include integration (an integrative approach to be open to both the home culture and host culture), separation (a rejection/alienation of host country culture and adherence to home country culture and co-nationals), and assimilation (an alienation from one’s own culture and an embracement of the host country culture) (5, 7, 8).

Numerous studies have demonstrated that unlike integration (which is generally considered the most effective coping strategy for international students against acculturative stress), separation and assimilation (which is considered to result in a moderate level of mental distress) (5, 8–13), international students who use marginalization as their coping strategy to handle acculturative stress are usually associated with the worst mental health outcomes, such as a high level of depression and anxiety, social phobia, and posttraumatic stress syndrome (14–17). However, limited empirical attention has been devoted to understanding the causing factors, psychological pathways, and other subtle differences of individuals who use this coping strategy. To address this gap, the present study focuses on Chinese international students who use marginalization during acculturation. For the purposes of this investigation, “marginalized Chinese international students” are defined as those who employ marginalization as their coping strategy during acculturation. This investigation seeks to elucidate the underlying narratives of these students, exploring factors associated with the use of marginalization as the coping strategy, what kind of international students are using this coping strategy, and the corresponding mental health outcomes.

### 1.2 Possible factors causing acculturative stresses for marginalized Chinese international students

Given that marginalized international students reject cultures from both host and home countries, which is rare compared with other three coping strategies, it is plausible that acculturative stress during their acculturation might come not only from the host-country side, but also from the home-country side. Extant studies have thoroughly examined the general acculturative stress for Chinese international students from the host-country side during their academic and social integration. Studies indicated that limited language-cultural proficiency and learning styles differences would lead to acculturative stress during academic and social acculturation (9, 18). This factor is particularly salient for those academically unprepared students who are involuntarily sent to overseas college by their parents (19). Additionally, during COVID-19, xenophobia and racial discrimination (20, 21), online learning challenges, safety and security concerns, financial strain, and the uncertainty caused by policy changes also emerged as key factors causing acculturative stress (22–24).

On the home-country side, there were multiple incidents happened domestically but could also potentially lead to Chinese international students’ rejection of home country when they were studying overseas. In 2012, when two Chinese international students were shot to death in their car during overseas staying, given that most Chinese international students are from upper middle/upper families (25, 26), domestic media coverage focused on the brand of the luxury car they were in when the incident occurred, which stirred public outcry not for the violence, but for the luxury lifestyle this population maintained (27). Also, because of the ideological conflicts between China and the West, Chinese international students who sided with the West were usually discriminated against: one female Chinese international student returnee during the outbreak of COVID-19 hotel quarantine was stigmatized as “giant infant” for disobeying quarantine regulation and asking for human rights (28, 29). Another example is that in 2017, one Chinese international student was slammed, stigmatized, and even doxed by Chinese netizens for giving a commencement speech praising the higher air quality in the United States than that of China (30, 31).

In addition to factors from both sides, their evolving transnational identities might also lead to acculturative stresses from both sides, including racial and gender identities. Given that Chinese society is relatively homogenous, their evolving gender and racial identity developed during the acculturation in the host country might be quite different compared to what they have been through in their previous years in China. To be more specific, because of the more traditional gender norms in China and the gender inequality in the Chinese workplace (32), Chinese male and female students might have different levels of social integration into the host country (33), therefore holding different views towards progressive agenda such as Diversity, Equity, Inclusion (DEI) programs in the West. Also, because the Western racial stereotypes and sexual racism have polarized views on Asian males and females (34–36), Chinese male and female international students might have various levels of social recognition, social support, and social capital, resulting in different levels of acculturative stress and social integration in the host country (33). For those who adhere to more traditional gender norms in China, or who are less favored by Western sexual racism, they might suffer from romantic rejection—a symptom describing the experience in which a person’s romantic advance is not reciprocated by a potential partner (37). In this case, marginalized Chinese international students’ romantic advances could be rejected by both potential Chinese and Western partners.

Furthermore, some of the Chinese international students might develop a “double dissident” political identity after their experiences in both the host and home country (38). Such an identity would make them skeptical towards both China and the West. However, due to fears of repercussions, they might withhold their critical views toward political figures or events. In addition to the commencement speech incident mentioned above (30, 31), another example is that a Chinese female international student’s Student Visa was revoked by the US government when she participated in a rally protesting against the US’ stance in the Middle East regional conflict (39). Seeing these repercussions, marginalized Chinese international students might feel excluded because of the “double dissident” mentality and the fear of repercussions from both sides, culminating in political depression–the process by which individuals absorb and internalize perceived political injustices, thus transforming their personal despair into a shared public affect that both undermines individual well‐being and shapes collective political discourse (40).

### 1.3 Prior studies on marginalized groups

There is limited extant literature on marginalized international students. One study on first-year undergraduate international students in the US suggested that there are two ways of marginalization, voluntary marginalization refers to willingly marginalizing themselves from peers (local and co-nationals) to focus on study, whereas involuntary marginalization usually denotes the forced exclusion or isolation of an individual from a group or society, often due to factors beyond their control, such as cultural differences, lack of social support, or inability to navigate the host culture effectively (41). Prior studies also indicated that marginalized groups often form counter-societies or counter-spaces with alternative value systems to gain recognition and validation (42–44).

In light of the development of the internet and social media, it is plausible that Chinese marginalized students might form counter-spaces to gain recognition and validation in online communities (42–44). Earlier studies on other marginalized groups such as women of color (45), the indigenous population (46), and the Latinx LGBTQ community (47) all illustrated that online counter-spaces are vital for marginalized groups, providing them with platforms for recognition, validation, and activism. However, excessive reliance on counterpaces such as online communities can lead to international students’ addictive disorders, because these online counter-spaces may foster a sense of belonging and community but can also reinforce isolation from the broader society, exacerbating feelings of marginalization and potentially leading to maladaptive behaviors.

### 1.4 Current study

Despite considerable research on acculturative stress among Chinese international students, few studies have focused specifically on those who adopt marginalization as their primary coping strategy. Prior literature has largely examined integration, separation, and assimilation (19, 48–50), with limited attention to the unique challenges and identity formations of students who reject both their home and host country cultures. This study fills that gap by exploring the distinct identity clusters and psychological pathways of marginalized Chinese international students, as well as the consequent mental health outcomes associated with their reliance on online communities as coping spaces. According to Self Determination Theory (SDT), humans have universal psychological needs for competence, autonomy, and relatedness (51), thus the possible formation of online spaces could reflect their psychological responses to satisfy these needs. Moreover, while the use of online platforms could serve as counter spaces for marginalized groups, it might also lead to various mental health outcomes. To delve further into the abovementioned questions, the study will pursue the following research questions:

1. What are the factors that contribute to the marginalization of Chinese international students
2. What are the possible psychological pathways for the formation of various identity clusters within online communities?
3. How do members of each identity cluster describe their mental health experiences?

## 2 Method

### 2.1 Data

The current study is a qualitative study based on interviews of 12 Chinese international students in an online community. The authors subscribed to a popular Chinese content creator who posts his videos on multiple online platforms (Chinese domestic and foreign platforms). Due to the extremely sensitive nature of the study, the user ID, the content, and the specific social media platform that the content creator uses will NOT be disclosed in an effort to protect the content creator as well as his followers from imminent threat and possible harms based on prior cases (39, 52). The researchers joined the online community owned by the content creator. To gain participants’ trust, the researchers engaged in community events such as discussions and interactions with community members. The online community is a comprehensive platform that offers venues for discussions about gaming, friending, politics, academic life, etc. After two months of immersive experience, the researchers posted a thread in this general discussion section, sharing the study’s purpose, informed consent, and definitions of marginalization based on Berry’s definition (the rejection of cultures from both the host and home nation) (5), as well as the inclusion criteria. The inclusion criteria were: 1) participants must be born and raised in China. 2) participants must be over 18 years old. 3) participants must currently hold a student visa studying in a foreign society, and 4) participants must be self-categorized as marginalization based on the given definitions. A total of 12 participants filled out and returned the informed consent to participate in the study.

The interview took place from May 2, 2024, to May 15, 2024. All interview durations ranged from 30-45 minutes. The interviews were conducted through the social media’s voice chat function in Mandarin Chinese. Semi-structured questions were used to probe possible factors leading to their marginalization, psychological pathways, and mental health outcomes. Pseudonyms were assigned to participants to safeguard their identity and ensure complete anonymity. The interviews were recorded and transcribed in Chinese. Because of the involvement of human subjects, the study received approval from the Institutional Review Board (IRB) of the first author’s institution.

### 2.2 Data analysis

All interview transcripts were imported into MAXQDA and analyzed using an abductive method of qualitative theme analysis, following the procedures outlined by (53). This analysis comprised four systematic steps: 1) The researchers thoroughly read each transcript to become deeply familiar with the interview data, setting the stage for the coding process. 2) To capture a wide range of semantic meanings and understandings of Chinese students’ marginalization experiences, the authors conducted two rounds of inductive coding on the raw data, ensuring a comprehensive capture of nuanced insights. 3) When multiple codes collectively represented a more profound central concept or phenomenon, these codes were grouped and categorized into subthemes and themes. 4) The authors then rigorously analyzed the identified (sub)themes by continuously comparing them to key concepts such as autonomy, competence, and relatedness of Self-Determination Theories (SDT). This comparative analysis facilitated the identification, annotation, and categorization of codes and themes pertinent to the study’s objectives. To strengthen the reliability of our findings, we conducted extensive participant observation within the online community over a two-month period, which enabled us to contextualize and corroborate the narratives obtained from the interviews. Furthermore, we checked and rechecked the transcribes with the participants to confirm that our emerging themes accurately reflected their experiences. The finalized themes, subthemes, codes, and quoted excerpts presented in the findings were translated from Chinese to English, and the first author verified the translations for accuracy to ensure valid data interpretation.

**Table 1** provides the demographic information of the participants.

**Table 1:** List of participants and demographic information.

Name	Age	Perceived Family SES	Self-reported Academic Performance	In Relationship	Subgroup	Years Abroad	Gender
P1	19	Upper	Low	Yes	Gamers	1	M
P2	18	Upper	Low	Yes	Gamers	1	M
P3	21	Upper-mid	Mid	No	Gamers	2	M
P4	20	Upper-mid	Low	No	Gamers	2	F
P5	21	Upper-mid	Mid	Yes	Gamers	2	F
P6	21	Mid	High	No	Dissidents	3	M
P7	22	Mid	Mid	No	Dissidents	4	M
P8	23	Upper-mid	High	No	Dissidents	1	M
P9	20	Mid	High	No	Dissidents	2	M
P10	22	Mid	Mid	No	Bachelors	3	M
P11	24	Mid	High	No	Bachelors	2	M
P12	23	Mid	Mid	No	Bachelors	5	M

## 3 Findings

### 3.1 RQ1: factors for marginalization

In general, there are three factors associated with Chinese international students’ marginalization and their participation in an online community: involuntary sojourning, romantic rejection, and political depression. In response, they chose online gaming, online pornographic use, and online political discourses as their specific coping strategies. Having said that, however, they all admit that they only partially reject, rather than fully denying the cultures of both the home and host nation.

#### 3.1.1 Involuntary sojourning—Gamers

Many expressed that involuntary sojourning is their main factor for marginalization and devotion in an online community. What is also noteworthy is that most who reported involuntary sojourning as the factor for marginalization were from an upper/upper-middle class family background, admitting they are the second-rich generation whose parents run family businesses and large corporates (54). These participants are categorized as Gamers. Gamers are not academically prepared and recorded poor academic performance during their domestic high school. Their studying abroad is involuntary, as their parents dominated the decision-making and sent them overseas to get a college degree. Therefore, they lack motivation, have low language-cultural proficiency, and couldn’t fit in the academic learning environment of the host country. As a result, their rejection on China side mainly stems from the authoritarian parenting style and the decision to send them abroad, while the rejection towards host country comes from the frustration of having low academic performance and loss of status. To cope with the associated mental distress, Gamers seek validation from achievements through online gaming. Both males and females suggested that online gaming could offer them a sense of achievement.

*I feel way much better when we play League of Legends and beat those dumb Laowai (foreign players) up than sitting in the classroom and getting bored. (P2).*

*I felt more confident when I played World of Warcraft. As a female player, I think everyone was nice to me. Perhaps when I graduate someday, I can be a streamer and make money out of it. (P5).*

#### 3.1.2 Romantic rejection—Bachelor

There are also several participants who suggested that the romantic rejection is the reason that led to their marginalization. These participants are categorized as Bachelors in this study. All Bachelors are males, and they reported a high level of low self-esteem due to perceived unattractive physical characteristics (such as low perceived facial attractiveness and low body height), as well as perceived lower family socioeconomic background compared with other Chinese international students. They also reported the highest adherence to traditional social/gender norms compared with other identity clusters, therefore holding an unfavorable view towards DEI programs in the West and female advocates of these programs. They are generally from middle-class families. Interestingly, some used to have romantic partners when they were in China. But after they went through a few romantic rejections during their overseas experience, they generally displayed social avoidance when interacting with Chinese as well as foreign females. So, their rejection toward China side mainly comes from their rejection of Chinese female co-nationals who they perceive as materialistic-driven or liberalized gender view holders. And their rejection of West is based on their negative experience with sexual racism and DEI programs. As a result, they use “*Diao Si”* (a Chinese internet slang with a derogatory connotation, referring to single male with low socioeconomic status) to describe themselves, admitting that they would watch female streamers streaming soft porn content or explicit pornography to mitigate their sexual impulse/intimacy needs.

*Girls are so picky around the globe and always love to hang out with the Gao Fu Shuai (an internet slang referring to the combination of the tall, the rich, and the handsome). I am a Diao Si and I wouldn’t spend so much money gifting girls only to be her secondary options. I’d rather use the money to tip the streamers at least my feedback is quite positive. (P10).*

*I used to laugh at a DEI admitted student who apparently is not at our level in the classroom, and I shared my opinion in my WeChat Moments in Chinese (an Instagram-like social media for sharing images and short videos). One Chinese female student who was on my WeChat contact, but we barely talk to each other, screenshot, translated, and reported my WeChat Moment post to the student affair and my academic advisor. For that I was admonished and lost my scholarship. I think the DEI-based admission is dumb and the girl reported me is a minion of the West and a traitor! (P12).*

#### 3.1.3 Political depression—Dissidents

Several participants also consider the factor of political depression as the reason for their marginalization. These participants are categorized as Dissidents in this study. They are all males who claimed to be politically depressed due to the perceived grievance for not being able to speak out. They are critical of China and Western governments, but due to factors such as media censorship and fear of repercussions, they could not express themselves freely online or in public spaces.

*My government is composed of corrupted government officials, colluding with the capitalist moguls to exploit the proletarians. But the host nation’s government is no way better. Look at how they are supporting war criminals in the Middle East regional conflict! Capitalists around the globe are the same demons that need to be purged. (P7).*

*A lot of Chinese international students are the “little pink” (a derogatory term to describe Chinese with strong nationalist sentiment). I don’t want to be one of them. But local people are not so much interested in Chinese politics. So, I choose to engage in political discourses with other netizens here to enjoy my free speech. (P9).*

### 3.2 RQ2: the diverse pathways behind identity clusters

#### 3.2.1 Psychological pathway of Gamers

Because of their second-generation rich identity, Gamers’ parents are mostly first-generation entrepreneurs who are known for their strongman leadership and authoritarian managing/parenting style in family business/family. It is also intuitive when the Gamers revealed that their family business is financially rewarding but time consuming for their parents, so gamers usually receive strong financial support but little emotional support from parents. That also said, they grow up in an environment where everyone is expecting them to be as successful as their first-generation rich parent(s). Therefore, they tend to be more competitive compared with other identity clusters.

*I was told that I should excel in every facet of life, just like my dad. I’m not sure if I can be that good, but I’ll try my best. (P3).*

Although enhancing their academic performance requires sustained effort, their favorable financial conditions enable them to experience success in pay-to-win gaming environments (55, 56). Additionally, as second-generation affluent individuals, they traditionally received preferential treatment during social interactions in China. However, while studying abroad, their limited academic abilities and language proficiency often prevent them from distinguishing themselves and receiving comparable preferential treatment, especially since many Gamers reside in less populated areas with few co-nationals. Consequently, gaming becomes a venue through which they can experience success, gain favorable treatment, and receive emotional support.

*One good thing about gaming is, when you have a lot of money, you can win and there are many people coming towards you, hoping you would use your power to help them win (P1).*

#### 3.2.2 Psychological pathway of bachelors

Bachelors are ideological conservatives who hold traditional gender views on society and family roles. Therefore, they generally evaluate females on the perceived femininity and appearance when seeking romantic partners. However, because their financial status is usually not as good as Gamers and other second-generation rich, and because their perceived less attractive physical traits and personalities, their romantic advancement towards more appealing female co-nationals usually failed to bear fruit.

*Whenever there were Chinese student gatherings, girls would circle around the rich guys and beg for attention. It’s disgusting and such a waste of time. (P11).*

Furthermore, also because of their traditional gender/racial roles, they feel detached from local DEI advocates, as well as co-nationals who are ideologically aligned with the progressive DEI agendas. This detachment further diminishes their chance of seeking romantic advancement towards co-national Chinese females and local females.

*Girls should look exactly like girls. I don’t like fat ladies who behave like men here and those Chinese girls who pretend to be having a white heart. (P12).*

Their traditional gender views and conservative ideology don’t resonate well with female co-nationals as well as the local community. However, on the internet and this online community, they can find others who know and share their pain. More importantly, they can seek virtual romantic relations with streamers who are in line with their traditional beauty standard with an affordable financial cost.

*Even if I tip just a small amount to streamers, they thank me wholeheartedly and do as I requested. But in reality, if you try to date a girl, this amount of money won’t get you anywhere. (P11).*

As a result, Bachelors form their identity cluster in the online community to find others who share their conservative gender views, bashing DEI policies, promoting conservative values, and using pornographic content to fulfill their sexual and psychological needs.

#### 3.2.3 Psychological pathway of Dissidents

Lastly, Dissidents in this study all indicate that they have strong interest in politics about China and the world. They identify themselves as academic overachievers who major in STEM subjects. Such a disparity between interest and major makes it hard to find local friends who can share their political passion. They are critical of Chinese government and policies, which alienated them from pro-government nationalist Chinese international students and those who are politically apathetic. Moreover, they disagree with host country government’s stance on certain regional conflicts, but the fear of repercussion from host country authority silenced their voice in public discourse and rallies. Therefore, they use this online platform to mitigate their political depression and voice their concerns freely with other community members.

*I can’t voice my opinions on domestic online platforms and my comments were always filtered or deleted in domestic social media. Now I can freely engage in discussions with the conservatives and liberals to discuss political issues, bypassing internet censorship and acquiring unbiased information, and that is my only purpose of being here. (P6).*

Furthermore, the more radical Marxist-Leninist-Maoist advocates in this cluster expressed a greater level of rejection toward both home and host country cultures. They disavow the current Chinese social reality as a socialist regime, asserting that it is colluding with ‘capitalist moguls’ to exploit workers. They also hold cosmopolitan views, claiming that all workers around the world should unite, thereby disapproving of the current nationalist sentiment on Chinese social media. They also detest the right-wing populist political movements and reject the importance of liberal democracy in the host country, emphasizing the significance of the ‘dictatorship of the proletariat.’ Therefore, their radical views usually lead to their exclusion from both home and host country public discourse.

*Only in online communities can I find those comrades who are ideologically pure and willing to fight and end the injustice. (P7).*

Their far-left political orientation also leads them to believe that sacrifice and violence are necessary to achieve greater good, which further marginalizes them from mainstream political conversations and renders them largely isolated in both societies. In this case, the online community serves as a crucial outlet for their views, offering a space where they can engage with like-minded individuals who share their ideological stance. This virtual space allows them to express their radical beliefs freely, without the immediate constraints of societal norms or the backlash they might face in real-world interactions.

*Chairman Mao used to say, “revolution is no dinner party”, so we should do whatever it takes to fulfill the greater good. (P9).*

### 3.3 RQ3: mental health experiences of each identity clusters

Participants described various experiences of emotional distress in relation to their coping strategies. For example, members of the Gamers cluster often mentioned preoccupation with gaming and reported feelings of irritability, anxiety, or sadness when they were unable to play or perform as expected (see P1 and P2’s quotes). Although these accounts are reminiscent of behaviors associated with problematic gaming, no formal assessment for Internet Gaming Disorder was conducted. Therefore, we interpret these narratives as indicative of gaming-related distress rather than a formal diagnosis of addiction.

*It’s okay for me to skip school, but it’s NOT okay when my gaming teammates need me, but I was unable to play. (P3).*

Bachelors, on the other hand, suggest feelings of inadequacy and isolation. These self-reported experiences, while suggestive of emotional difficulties, should be understood as personal perceptions of distress rather than definitive evidence of clinical depression or anxiety.

*Trust in your money, not your girls—that’s what I and my friends concurred in this community. Someday when you have money, you don’t worry about finding girls, they will find you. Now I don’t have the money, so no need for me to waste time interacting with them now. (P10).*

For the Dissidents cluster, several participants expressed a sense of political depression, social alienation, and frustration stemming from an inability to voice their views openly due to censorship and fear of repercussions. Although some accounts included references that might be interpreted as suicidal thoughts, these expressions are based on personal reflections and are not supported by formal clinical assessments. Thus, they are best understood as subjective reports of distress related to their political experiences.

*When society gives us no hope, exploiting us with real estate mortgages and car loans, when inequality has been cemented upon our births, when we have to work 996 (referring to 9am-9pm, 6 days a week), why should we sell our labor so cheaply to these capitalists? They want us to have kids so they can have more slaves in the future? We give them NONE! They want to use our labor to further accumulate capital? Then suicide is an option to fend off their exploitation and an ultimate way to show our rebellion. (P8).*

## 4 Discussion

The current qualitative research based on 12 marginalized Chinese international students provides insights into a heavily understudied group experiencing severe mental distress. Through semi-structured interviews, the study identified involuntary sojourning, romantic rejection, and political depression as primary factors contributing to their marginalization. Accordingly, three identity clusters were identified: Gamers are those who use online gaming as their coping strategy. Bachelors refers to Chinese international students who frequently use online communities for pornography use and conservative discourses. Lastly, Dissidents are a group of students who are critical of governments of both home and host country, so they use online communities to discuss political topics. Because of their unique familial socioeconomic status, academic performance, and personal emotional state, their identity clusters are stable and mutually exclusive. Lastly, all identity clusters were associated with mental health risks, with Gamers being at risk for Internet Gaming Disorder (IGD), Bachelors being associated with low self-esteem, depression, social alienation, and social withdrawal, and Dissidents being linked to severe anxiety, depression, and even suicidal ideations.

### 4.1 Discussion of RQ1 findings

The marginalization of Chinese international students in this study can be traced to three main factors: involuntary sojourning, romantic rejection, and political depression. Each of these factors significantly contributes to their sense of alienation and psychological distress during their overseas experience. As they navigate these challenges, students adopt a range of coping strategies that include online gaming, pornographic content consumption, and political discourse, primarily through online communities.

The involuntary sojourning as a factor for marginalization is consistent with prior studies (57–61). In this study, the Gamers reported involuntary sojourning as a major factor contributing to their marginalization. Many were sent abroad by their upper or upper-middle-class parents, who decided that they should pursue higher education overseas. As a result, the Gamers felt a lack of autonomy, which led to disengagement from both academic and social settings in their host country. The pressure to succeed, combined with low academic performance and language-cultural proficiency issues, diminished their sense of competence. They also lost relatedness, as they were geographically distanced from their home community and could not connect meaningfully with peers in their host country. To cope with these stresses, Gamers turned to online gaming as a means to restore their sense of competence and autonomy. Gaming provided them with an outlet to achieve success, where their financial resources enabled them to excel through Pay-to-Win features (55, 56). This success in the virtual world allowed them to gain recognition and favorable treatment, fulfilling their need for relatedness in an online community. Thus, online gaming served as a critical coping strategy, providing the Gamers with a temporary sense of mastery and control.

The Bachelors faced romantic rejection as a major source of marginalization. Their experiences of rejection from both Chinese and local females were largely driven by perceived differences in physical attractiveness and socioeconomic status compared to other students. This phenomenon is consistent with prior studies on the gender differences of Chinese international students’ acculturation strategies in Western countries (33, 48). Due to their lower financial standing and perceived less attractive physical traits, they felt incompetent in romantic and social contexts, leading to increased social isolation. Their traditional gender norms, which were strongly ingrained in their beliefs about masculinity and femininity, further alienated them from both local and co-national peers, particularly those who supported DEI policies. The Bachelors’ disillusionment with romantic prospects led them to adopt online communities as a source of social validation. By engaging in online pornographic content and interacting with streamers, they sought to fulfill their needs for autonomy and competence. The ability to tip streamers and gain a sense of power and attention in these online spaces gave them a temporary sense of control over their romantic and social exclusion.

For the Dissidents, political depression stemmed from their critical views on both the Chinese government and the host country political stance. Their dissatisfaction with the political landscapes in both countries is documented in a prior study (38). Such a “double dissident” political identity contributed to a sense of powerlessness and alienation, particularly as they felt unable to express their views freely due to censorship and fear of repercussions. As a result, the Dissidents turned to online political discourses as a means to engage with others who shared their political views. These discussions provided a platform for them to express their critical opinions and engage in debates about capitalism, socialism, and global issues. These online interactions helped them fulfill their need for autonomy and relatedness by connecting with like-minded individuals, though they remained isolated from both their home country’s nationalists and the host nation’s political mainstream.

### 4.2 Discussion of RQ2 findings

The experiences and psychological pathways of Gamers can be understood through SDT, which highlights the importance of fulfilling psychological needs for autonomy, competence, and relatedness. Their lack of autonomy comes from the parental decision to send them abroad, which reduces their motivation and engagement in both academic and social settings in the host country. In addition, the lack of competence is reflected by their inability to improve their academic performance. The involuntary sojourning also cuts their ties with domestic social circles, causing a loss of relatedness. On the other hand, gaming provides them with a sense of control and self-direction, fulfilling their need for autonomy. Also, considering many of the Pay-to-Win features and game designs in online gaming (55, 56), then their financial capabilities under this mechanism would ensure their wining in the virtual world, as well as friending requests from other players who long for their power in the game, which would fulfill their need for competence and relatedness. Furthermore, their eagerness for parental recognition as second-generation rich is also in alignment with prior studies (54).

For Bachelors, their marginalization stems from their failure to advance their romantic relations towards females of co-nationals and local nations due to various reasons. On the racial stereotype end, it has been widely reported that White females tend to exclude Asian males sexually, while Asian females are generally preferred by other ethnic groups due to perceived femininity and alignment with traditional gender roles (57, 58, 60). Therefore, the male competition for female Chinese international students heightened, as local nationals, second-generation rich international students would compete with Bachelors for female Chinese international students’ attention. When Bachelors lose the competition, they tend to equate successful romantic relationships with a combination of physical attractiveness and financial superiority, downplaying or neglecting the importance of personality and individual differences (See P10, P11, and P12’s quotes). Because of their failure in the competition, they also suffer from a loss of competence. On the cultural end, because they are more culturally conservative and look for a male dominance in a romantic relation, they don’t appreciate females with progressive gender views and independent personalities. In this line of thinking, paradoxically, should they lower their standard and embrace liberal gender norms, they would also lose their “autonomy” and male’s pride. Furthermore, the host country DEI policies and culture would also alienate them for their conservative-leaning ideology, for which they also detest local feminists or Chinese females aligning with Western feminist ideas (see P12’s quote). On the other hand, through online pornographic use, they seek autonomy and competence in romantic relations through tipping streamers. This approach to revive their autonomy and competence is also deemed more cost-effective (see P10 and P11’s quotes on tipping streamers). Furthermore, they could also achieve relatedness when they find other ideologically conservative co-nationals on the online community. The misogynistic views Bachelors expressed, along with the perception that women are materialistic and objectified (See P10, P11, P12’s responses), resonated well with the larger INCEL (involuntary celibates) community in the West, whose members are also notorious for women-hating and suffer from acute mental issues and violent tendencies (62–65).

Lastly, Dissidents perceive themselves to be deprived of autonomy and relatedness in real life due to their critical views on both home and host nation, as well as the fear of repercussions (31, 39, 52). In the online community, Dissidents achieve these psychological needs via freely expressing their political ideas and finding “comrades” who share their radical views. Additionally, the study offered new insights into the current literature on Chinese international students’ political identity. Namely, prior studies conclude that Chinese international students can be categorized as nationalists, globalists, and pro-democracy liberalists (66–68), yet the current study suggested that in addition to these political identities, radical left-leaning, Marxism/Leninism/Maoism oriented political identity is burgeoning among Chinese youth. This finding is intuitive in the post-COVID 19 context, given the gradual centralized Chinese government and nationalism-leaning diplomatic stance (69–71), the stumbling economy, high unemployment rate, and exploitative workplace culture (72–75). Although prior studies on Chinese international students’ patriotism suggested that their overseas study led to a heightened national identity and pro-government stance (76, 77), this study findings suggested otherwise.

### 4.3 Discussion of RQ3 findings

The qualitative data suggest that the coping strategies adopted by marginalized Chinese international students are associated with varied self-reported mental distresses. For instance, Gamers described how their engagement in online gaming fostered a sense of belonging and provided opportunities for social validation (78). At the same time, several participants indicated that interruptions in their gaming routine were linked with feelings of sadness or anxiety. Although these accounts resemble symptoms noted in research on problematic gaming behavior (79), our findings are based solely on subjective reports rather than on standardized clinical evaluations.

Similarly, the narratives from Bachelors indicate that repeated experiences of romantic rejection and perceived social inadequacy may contribute to their reported feelings of isolation and distress. Their discussions suggest that challenges in securing satisfactory romantic relationships may reinforce negative self-perceptions. However, these self-reported experiences should be interpreted as subjective indicators of distress, rather than as evidence of clinically significant depression or anxiety.

For the Dissidents, the accounts reflect a deep-seated sense of political frustration and social alienation. Participants in this cluster described feelings of powerlessness and, in some cases, mentioned thoughts that could be construed as suicidal. Given that these reflections were self-reported and not measured with clinical instruments, they should be regarded as personal expressions of distress related to political and social disenfranchisement.

In sum, while our findings highlight a range of self-reported emotional challenges among the three identity clusters, they do not provide clinical diagnoses. These results underscore the need for further research that incorporates quantitative and clinical methodologies to better understand the prevalence and severity of these experiences. Future studies could clarify whether these self-reported symptoms reach clinical thresholds, and thereby inform the design of culturally sensitive support services for marginalized international students.

### 4.4 Implications

The findings of this study offer significant insights into the mental health and well-being of marginalized Chinese international students, with implications for policy, practice, and future research.

Firstly, universities should develop targeted, culturally sensitive mental health support services. This could include training faculty and staff to recognize signs of distress unique to marginalized students, creating peer support groups, and offering workshops on adaptive coping strategies. Collaboration with community organizations that specialize in mental health could further enhance these services.

Secondly, mental health professionals should work to provide accessible counseling and intervention programs tailored to the specific experiences of international students. This might involve offering services in Mandarin and ensuring that interventions consider the unique cultural and acculturative challenges faced by these students. Professionals could also pilot online counseling platforms or moderated online support groups, which may be particularly effective given the students’ reliance on digital communities.

Thirdly, online communities themselves can play a proactive role in supporting marginalized students. Administrators of such platforms should consider establishing dedicated spaces or forums for mental health support, where trained moderators or peer counselors can offer guidance, share resources, and foster a sense of belonging. These virtual safe spaces can help bridge the gap between the students’ online and offline lives, mitigating feelings of isolation.

Collectively, these recommendations suggest that a multi-level approach involving universities, mental health practitioners, and online community managers is essential for addressing the challenges identified in this study. Future research should explore the effectiveness of these interventions in reducing acculturative stress and improving overall mental health outcomes among marginalized Chinese international students.

## 5 Conclusion

This study provides a nuanced understanding of how marginalized Chinese international students navigate acculturative stress and identity formation through distinct online coping strategies. Our findings contribute to broader discussions on the mental health challenges and identity dynamics among international students by highlighting that marginalization as a rarely seen coping strategies can exist in online spaces. While this coping strategy can offer autonomy, relatedness, and competence to community members, it could also lead to negative mental health outcomes that need further investigation. Practically, the study filled the research gap, helping universities, mental health professionals, and online community moderators understand and realize the urgent need to develop culturally sensitive support systems and safe spaces that foster open dialogue and emotional well-being. By tailoring interventions to address the unique experiences of these students, institutions can help improve academic performance, enhance social integration, and ultimately promote healthier identity development among international student populations.

## 6 Limitations

This study, while providing valuable insights into the experiences of marginalized Chinese international students, has several limitations that should be acknowledged. The present study’s qualitative approach allowed us to capture nuanced narratives about identity formation among marginalized Chinese international students. However, the sample size (N=12) and the recruitment from a single online community may limit the generalizability of our findings to the broader population of Chinese international students. While qualitative research is not primarily aimed at generalizability but rather at gaining in-depth insights, we acknowledge that our sample may not fully represent the diversity of experiences within the larger community. Moreover, although we employed abductive thematic analysis and engaged in member checking and extensive online observation to triangulate data, these efforts could be further strengthened by incorporating multiple data sources (e.g., additional online communities, offline interviews, or observational data) in future research.

In addition to the relatively small sample size and limited diversity of participants, our study is cross-sectional in nature, capturing only a snapshot of the experiences of marginalized Chinese international students at one point in time. Given that identity formation is a dynamic process, a longitudinal design could provide deeper insights into how these students’ identities, coping strategies, and mental health outcomes evolve over time. Future research should incorporate longitudinal methods to explore the temporal shifts in acculturative stress and identity development, thereby offering a more comprehensive understanding of these processes.

## Data Availability

The datasets presented in this article are not readily available due to the sensitive nature of the study, and the data supporting its findings will not be shared or disclosed. Requests to access the datasets should be directed to ruiningjin@gmail.com.
